# Sinusoidal LED light recipes can improve rocket edible biomass and reduce electricity costs in indoor growth environments

**DOI:** 10.3389/fpls.2024.1447368

**Published:** 2024-10-03

**Authors:** John D. Stamford, Tanja A. Hofmann, Tracy Lawson

**Affiliations:** School of Life Sciences, University of Essex, Colchester, United Kingdom

**Keywords:** LED lighting, lighting regimes, photosynthesis, electron transport, diurnal, energy saving

## Abstract

Accumulation of edible biomass by crop plants relies on maintenance of a high photosynthetic rates across the photoperiod, with assimilation rate (*A*) generally responding to increasing light intensity in a hyperbolic fashion. In natural environments light fluctuates greatly over the course of the day, however in Controlled Environmental Agricultural (CEA) systems, light intensity can be supplemented or precisely controlled using LEDs to create near optimum conditions. In such indoor growth environments light is often delivered as a square wave and recommendations to horticulturalists are given in the form of Daily Light Integrals (DLI). However, this does not take into account the slow photosynthetic induction at the start of the photoperiod and the decline of *A* towards the end of the photoperiod, which has been demonstrated by several previous studies. Square wave light regimes therefore potentially cause suboptimal photosynthetic utilization of the applied lighting and waste electricity. Here we have adapted light recipes to gradually increase and decrease in intensity to take account of these findings. We demonstrate that, utilising a sinusoidal light regime capped at 250 μmol m^-2^ s^-1^, it is possible to increase edible biomass of rocket (by ca. 20%) compared to square wave delivered at 250 at the same DLI. Additionally, this can be achieved using less electricity (0.6%), therefore reducing energy costs and improving profitability. We suggest that capping maximum light intensity at 250 µmol m^-2^ s^-1^ improves the operating efficiency of PSII photochemistry (*Fq’/Fm’*) also known as the photosynthetic efficiency by maintaining *A* later in the photoperiod. We show that a higher electron transfer rate (ETR) is maintained in these treatments over the photoperiod compared to higher light intensity caps, resulting in a greater Daily Photochemical Integral (DPI). We attribute this to less NPQ due to a greater sink capacity for the end products of electron transport, ATP and NADPH, as *A* is kept high for longer.

## Introduction

For crops to accumulate edible biomass, they must maintain high net photosynthetic rates (Zelitch, 1982), which are largely dependent on the quality and quantity of light (photons) received by the plant. In natural environments, light can fluctuate greatly across the course of a day (e.g. due to periodic cloud cover creating sun and shade flecks) and photosynthetic carbon gain can therefore intermittently drop significantly, impacting on the cumulative rate over the growing season and thus overall yield (Sinclair and Muchow, 1999). Controlled Environment Agricultural (CEA) systems are becoming increasingly important to ensure sustainable food production (Shamshiri et al., 2018), especially in the face of climate change, growing global population sizes and expanding urbanisation (Foley et al., 2011). In some CEA systems, such as greenhouses, natural light can be supplemented artificially, in others, such as vertical farms, light may be controlled entirely, for example by using LED lights to create near-optimum conditions (Stamford et al., 2023). LEDs confer many advantages over more traditional lighting in horticultural applications, as they can be precisely adjusted in real-time via sensors and control systems to create adaptive lighting that maintains light intensity to pre-determined thresholds (van Iersel and Gianino, 2017) as well as enabling specific wavelengths to be tailored to growth requirements (see reviews Stamford et al., 2023).

Photosynthesis relies on light energy driving the rate of electron transport (ETR) and electron flow through photosystem II (PSII) and I (PSI), resulting in the production of ATP and NADPH in the light dependent reaction (LDR) and the rate of CO_2_ fixation or carbon assimilation in the Calvin Benson Cycle. The relationship between light intensity and photosynthetic rate (*A*) (measured as μmol CO_2_ m^-2^ s^-1^) typically follows a hyperbolic response, with a linear increase from darkness as light intensity increases up to a certain level (depending on the crop species and growing conditions) after which photosynthesis begins to saturate and further increases in light do not result in higher rates (Parson et al., 1998). Therefore, setting lighting intensity thresholds above saturation would not optimise photosynthesis, and costly electricity may be utilized for no gain in edible biomass.

Photons absorbed by the pigments in PSII have one of three competing fates; they are either: 1) utilised for the LDR (photochemical quenching) driving *A*, 2) dissipated as heat (non-photochemical quenching/NPQ) or 3) re-emitted as chlorophyll fluorescence (Baker, 2008). Measurements of chlorophyll fluorescence provide a non-destructive and rapid assessment of the operating efficiency of PSII photochemistry (*Fq’*/*Fm’*), which can be used to determine ETR and used as a proxy for *A* (Murchie and Lawson, 2013). Intensities exceeding light saturation result in increases in the rate constant for NPQ, as a way to dissipate excess photons, and correspond to a decrease in the quantum efficiency of photosystem II (PSII) photochemistry (*Fq’*/*Fm*’) (Elkins and van Iersel, 2020a, Palmer and van Iersel, 2020). Although vital for photo-protection, the slow reversal of NPQ processes can limit photosynthetic carbon gain, particularly in dynamic light environments (Kromdijk et al., 2016; Long et al., 2022). Further increases in light intensity, in which NPQ is insufficient to quench excess photons, can lead to damage of the photosynthetic apparatus known as photoinhibition (Baker, 2008; Murchie and Lawson, 2013) and a significant drop in photosynthetic rate. Recovery from photoinhibition can be considerably longer than reversal of NPQ, taking days which leads to a greater cumulated decrease in *A* with implications for biomass accumulation.

Several studies have reported a drop in photosynthetic assimilation rate (*A*) towards the end of the photoperiod in a number of species (Sawannarut et al., 2023; Kaiser et al., 2015; Matthews et al., 2018), with some demonstrating a reduction in *A* equivalent to 20% of the predicted total daily carbon assimilated (Vialet-Chabrand et al., 2017), potentially greatly impacting on crop growth/yield and/or utilizing unnecessary energy. Reductions in assimilation rate toward the end of the photoperiod (which are most likely species specific) result in a decrease in sink capacity for the end products of the Electron Transfer Chain (ATP and NADPH) (Harbinson et al., 1990; Ghildival, 1991; Paul and Pellny, 2003; Murchie and Lawson, 2013; Fabre et al., 2019). It has been suggested that sugar accumulation over the photoperiod could provide a feedback mechanism reducing CO_2_ demand by impacting on photosynthetic gene expression (Jang and Sheen, 1994; Paul and Pellny, 2003). Sawannarut et al. (2023) also reported that the speed of photosynthetic induction decreased in the afternoon, possibly due to lower stomatal conductance values limiting *A* or due to changes in the ratio of Rubisco to Rubisco activase later in the photoperiod (Mott and Woodrow, 2000). Slow photosynthetic induction when plants are transferred to high light or at the start of the photoperiod causes wasteful ‘foregone’ *A* (Long et al., 2022) that should be considered alongside the afternoon drop in *A* to reduce unnecessary electricity expenditure at the start of the photoperiod in indoor growth environments.

The total light received by the plant over the course of the day, is known as the Daily light integral (DLI), and is a parameter that is frequently recommended to guide light regimes in CEA systems (Palmer and van Iersel, 2020; Bugbee, 2017; van Iersel, 2017; Nelson and Bugbee, 2014), however DLI is normally delivered as a square-wave regime where a specific photosynthetic photon flux density (PPFD) is maintained over a set time, without dynamically adjusting light intensity over the photoperiod to match the crop’s requirement to maintain high photosynthetic efficiency. However, as mentioned above, several studies have reported decreases in both photosynthetic efficiency and carbon assimilation in the latter part of the photoperiod under both square and dynamic fluctuating light regimes (Vialet-Chabrand et al., 2017; Matthews et al., 2018). These observations suggest that maintaining a set lighting target over the entire photoperiod may not be optimal for photosynthesis and plant growth or energy demands. There have been attempts to improve indoor crop growth by delivering a specific DLI at a lower PPFD over a longer photoperiod, with promising results in some crops where *Fq’*/*Fm’* was improved and edible biomass increased (Elkins and van Iersel, 2020a; Palmer and van Iersel, 2020; Weaver and van Iersel, 2020).

Optimizing indoor lighting regimes to more closely match the requirements of the crop in order to maximize photosynthetic demands, could improve productivity and marketable yield whist reducing energy input costs, thus increasing profitability. We hypothesized that the slow rise in photosynthesis at the start of the photoperiod and the drop in *A* towards the end of the photoperiod, could be overcome by adjusting PPFD from the commonly used square wave pattern to a sinusoidal light regime, i.e. in which there was one oscillation or period per day, increasing and decreasing intensity gradually, thus accounting for slow photosynthetic induction and reducing the need for photoprotective mechanisms. These regimes following the typical natural diurnal rhythm of illumination in the natural environment and should not be confused with studies that use oscillating light to probe photosynthetic processes (e.g. Lazár et al., 2022b). Here we have tested sinusoidal light regimes that either peak in the morning or the afternoon and examined the impact on biomass, physiology and energy use.

## Materials and methods

### Growth conditions

**Figure 1:**
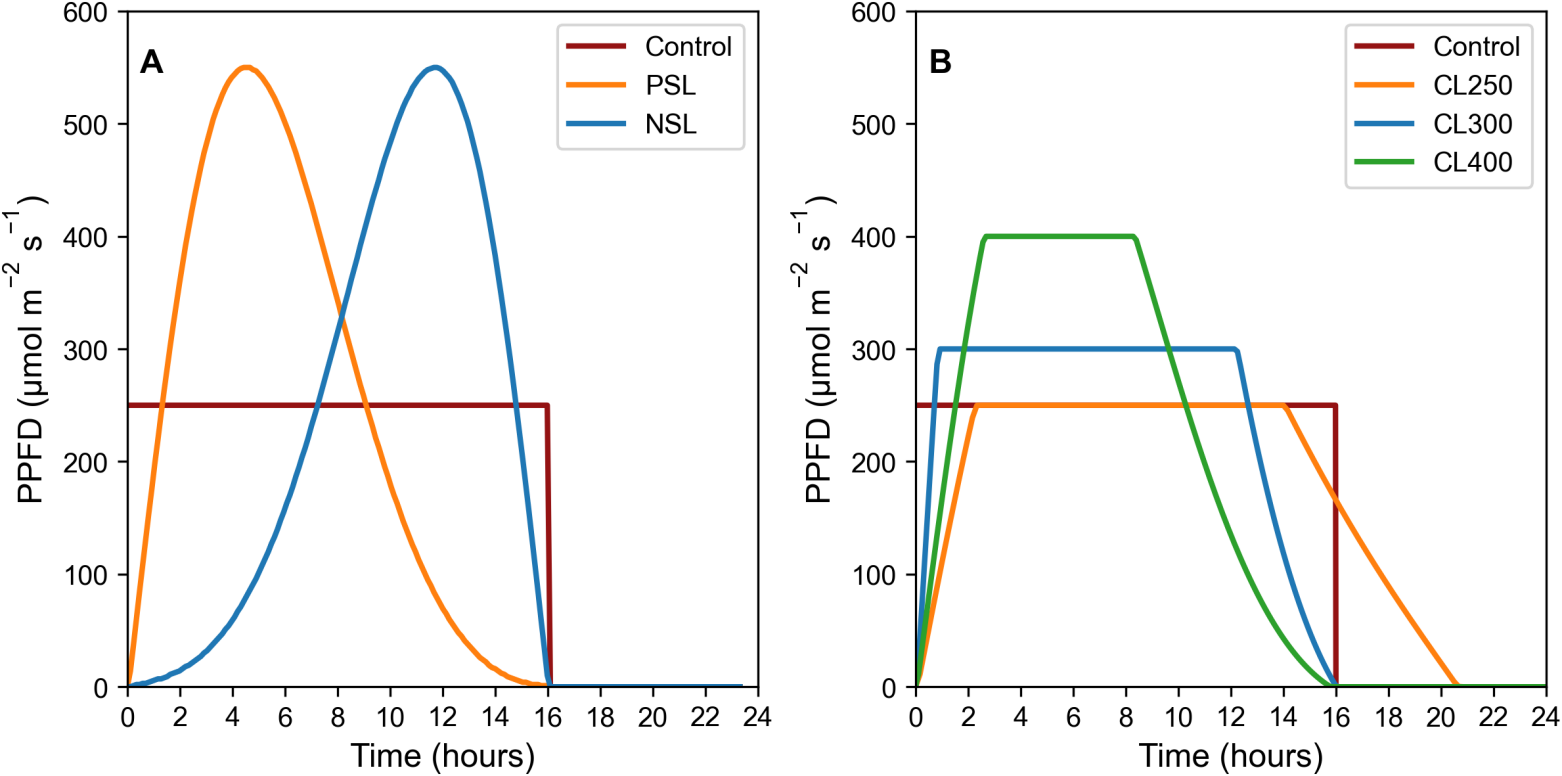
SL regimes **(A)**, LED light regimes used: Control 16 h square wave at 250 μmol m^-2^ s^-1^. PSL and NSL regimes 16 h light with peak PPFD of 550 μmol m^-2^ s^-1^; CL light regimes **(B)**, LED light regimes used: Control 16 h square wave at 250 μmol m^-2^ s^-1^. CL300 and CL400 both 16 hr light regimes with peak PPFD of either 300 or 400 μmol m^-2^ s^-1^ respectively. CL250 with a longer photoperiod of 21 h with a peak PPFD of 250 μmol m^-2^ s^-1^ in order to maintain the same DLI. All light regimes (SL and CL) have a DLI of 14.4 mol m^-2^ d^-1^. Plants were maintained at 21°C air temperature, 22°C under illumination, and a relative humidity of 70% ± 10%.

Rocket (*Eruca sativa*) cv. Sweet Intensity (Elsoms Seeds, Lincolnshire, UK) were sown in 14 cm tall and 5 cm wide pots in Levington Advance FS2+S soil (ICL Professional Horticulture, Suffolk, UK). All plants were grown in Fitoclima controlled growth rooms (Aralab, Rio de Mouro, Portugal) at 20°C, with humidity level set to 70% ± 10%. Lighting was provided by Heliospectra DYNA lamps (Heliospectra, Gothenburg, Sweden). Temperature levels under the lamps at plant height was 21°C. All experiments included a border row around each experimental block of plants to ensure plants were not influenced by being positioned at the edge of a block. Light regimes consisted of a control square-wave pattern (SW) at 250 μmol m^-2^ s^-1^, two sinusoidal light (SL) patterns (negative = NSL and positive = PSL), as well as three capped light (CL) regimes at 250, 300 and 400 μmol m^-2^ s^-1^ (CL250, CL300 and CL400) (for details see **Figures 1A, B**).

### Experimental design

#### Sinusoidal light regimes

Two sinusoidal light regimes were designed using Equation 1 (Equation 1), one with a positive skewed sinusoidal (PSL) and one with a negative skewed sinusoidal (NSL), both with a peak in light intensity of 550 μmol m^-2^ s^-1,^ and a DLI of 14.4 mol m^-2^ d^-1^(**Figure 1A**). The control treatment consisted of a square wave light regime for 16h at a constant light intensity of 250 μmol m^-2^ s^-1^, and with an identical DLI.


(1) $$y(t)\;=\;A\text{ sin}(\frac{t}{T\frac{\pi }{2}}\mathrm{skew}\cdot \text{sin}(\frac{t}{T\frac{\pi }{2}}))$$

Where *A* is the amplitude of the peak values, *T* is the period, skew is the skew factor, *t* is the time.

#### Capped sinusoidal light regimes

A second experiment was conducted in which the light regimes outlined above and in **Figure 1** were modified by reducing the maximum light intensity. Three positively skewed sinusoidal light regimes were designed, with the maximum light intensity achieved capped at either 250, 300 or 400 μmol m^-2^ s^-1^(CL250, CL300, CL400) (**Figure 1B**). Negative sinusoidal regimes were not considered as no additional benefit was observed in the experiments above and based on literature showing that photosynthesis is usually greater earlier in the photoperiod (Sawannarut et al., 2023; Kaiser et al., 2015; Vialet-Chabrand et al., 2017; Matthews et al., 2018). In order to ensure the same DLI for all light regimes of 14.4 mol m^-2^ d^-1^ the photoperiod was extended to ca. 21 h for the 250 μmol m^-2^ s^-1^ capped regime. A square wave lighting regime at 250 μmol m^-2^ s^-1^ and a 16 h photoperiod was again used as a control.

### Physiological and biomass measurements

Chlorophyll fluorescence measurements were collected using four Walz Micro-PAM heads (HeinzWalz, Effeltrich, Germany) over a 23 h period. Replicate measurements were gathered from eight plants over a 10 d period. Each Micro-PAM head was swapped between treatments to account for possible variability between measurement heads.

Before the light regimes were initiated, a dark adapted measurements of minimal fluorescence (*F_o_*) was captured followed by a saturating pulse to determine the maximum fluorescence in the dark (*F_m_*). These were used to calculate the maximum quantum efficiency of PSII photochemistry (*F_v_/F_m_* = (*F_m_ – F_o_*)/*F_m_*). During the light period measurements were captured at regular 20-min-intervals, of steady state fluorescence (*F*’) and maximum fluorescence in the light (*F_m_’*) and used to determine the operating efficiency of PSII, calculated using the following equation *F_q_’/F_m_‘* = (*F_m_’ – F’*)/*F_m_’* (Murchie and Lawson, 2013). The operating efficiency of PSII photochemistry (*F_q_’/F_m_‘*) *(* Genty et al., 1989) is the product of *F_q_’*/*F_v_’* and *F_v_’*/*F_m_’* in which *F_q_’*/*F_v_’* is the level of photochemical quenching of PSII and *F_v_’*/*F_m_’*, the maximum efficiency in the light adapted state, with any decrease in this value used as an indication of increased contribution of nonphotochemical quenching (Baker, 2008; Murchie and Lawson, 2013). *F_q_’*/*F_v_’* and *F_v_’*/*F_m_’* were derived from dark and light adapted measurements using the following formula *F_q_’*/*F_v_*’ = *F_m_*’-*F’*/*F_m_’*-*F_o_’* and *F_v_’*/*F_m_’* = *F_m_’* – *F_o_’*/*F_m_’*. *F_o_’* was calculated following the method of Oxborough and Baker (1997). Electron Transport Rate (ETR) was estimated using the following equation, ETR = *F_q_’/F_m_’* * PPFD * 0.84 * 0.5 (Maxwell and Johnson, 2000). Daily Photochemical Integral (DPI) was calculated by integrating ETR over a 24 h period.

Photosynthetic carbon assimilation (*A*) was measured as a function of light intensity (light response curves) using a LI-COR 6800 standard 6 cm^2^ cuvette with an integrated light source consisting of 10% blue and 90% red light (LI-COR, Nebraska, USA) with CO_2_ concentration set to 400 μmol mol^-1^, temperature set to 21°C, flow rate of 300 μmol s^-1^, and relative humidity set to 60%. Plants were kept at a light intensity of 1500 μmol m^-2^ s^-1^ until *A* was stable (approx. 10 – 15 minutes). Light intensities for the light response curve were 1500, 1300, 1100, 900, 700, 550, 400, 250, 150, 100, 50, 0 μmol m^-2^ s^-1^. The waiting time at each light level was a minimum of 30 s up to 180 s. These measurements were taken twice, 4 hours and 12 hours into the photoperiod. It should be noted that stabilizing *A* at 1500 μmol m^-2^ s^-1^ could induce some photoinhibition, reducing *A*.

Plants were harvested after 30 d of growth by cutting 1 cm above the base of the soil. Leaves were immediately placed onto a balance (<30 s from harvesting) to determine fresh mass. Dry masses were obtained after a minimum of 3 d in an oven set to 60°C.

### Statistical analysis

Analysis of Variance (ANOVA) was used to assess the differences between the means of three or more independent (or dependent) groups. Where data were not normally distributed, Kruskal Wallis (non-parametric) tests were used for comparisons. If the Kruskal-Wallis test indicated significant differences, pairwise comparisons between groups were subsequently performed using the Mann-Whitney U test, suitable for comparing the distributions of the groups. All statistics were performed with Python and the SciPy libraries.

## Results

### Sinusoidal lighting regimes

**Figure 2:**
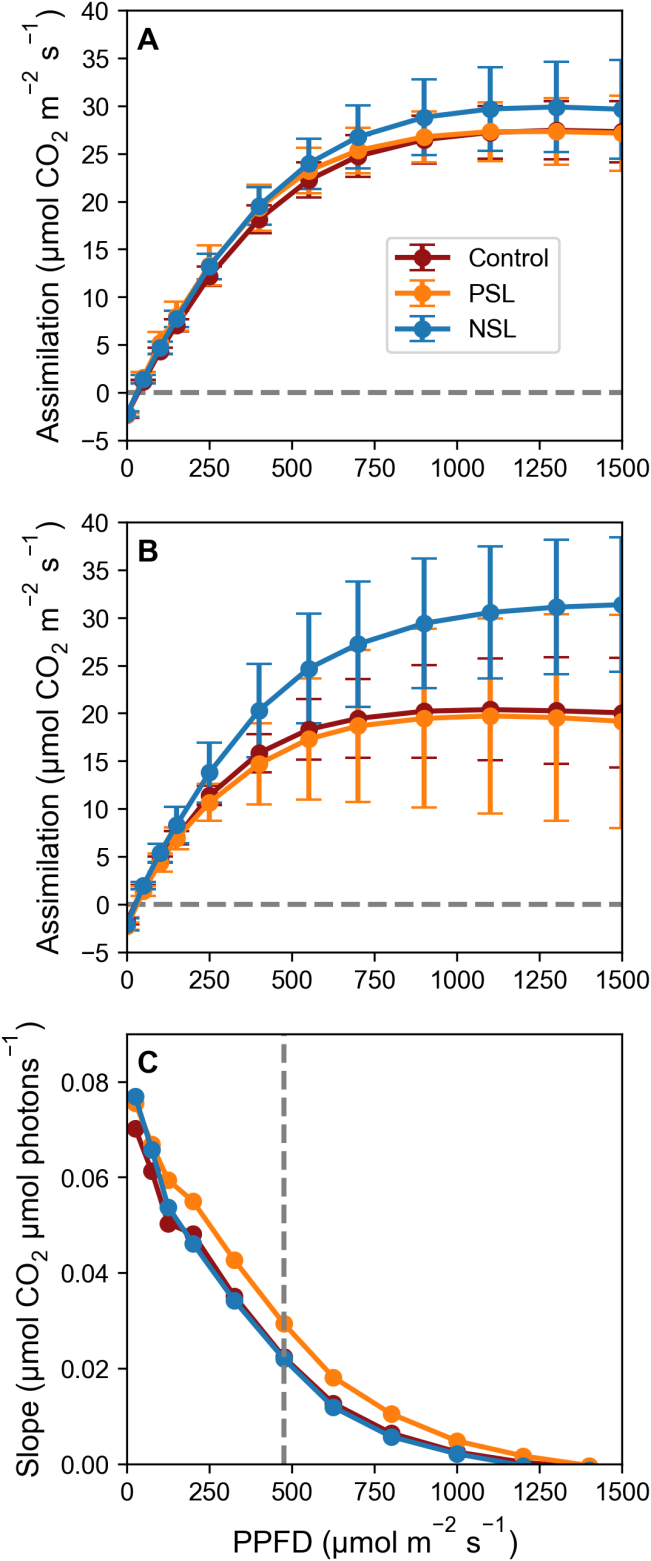
Photosynthesis **(A)** as a function of increasing PPFD [(0-1500 μmol m^-2^ s^-1^; *A*/*Q* curves]; **(A)** collected 4 h into the light regimes and **(B)** collected 12 h into the light regimes. CO_2_ concentration was maintained at 400 μmol m^-2^ s^-1^, leaf temperature at 21°C and RH at 65%. Dashed horizontal line (–) indicates an assimilation of 0 mol m^-2^ s^-1^. Error bars ± SD (n=5-9, except for evening NSL which was n=3). **(C)** The slope of the line between each pair of data points of **(A)**, plotted against the average light level of the two data points. Dashed vertical line (–) at 475 μmol m^-2^ s^-1^indicates the point at which the slope between datapoints visually starts to decline.

The photosynthetic capacities of plants under different light treatments were assessed by measuring photosynthetic carbon assimilation (*A*) across a range of light intensities (0 to 1500 μmol m^-2^ s^-1^) in both the morning and afternoon. As anticipated, the light response curves exhibited the typical hyperbolic pattern, showing a linear increase in initial response with rising light intensity (indicative of quantum efficiency), followed by a saturation of photosynthesis at ca. 475 µmol m^-2^ s^-1^ across all treatments and time points. Above this saturation point, photosynthetic rates remained relatively stable with increasing PPFD (**Figures 2A, B**).

In the morning (after 4 hours in the light), no discernible differences in quantum efficiency were observed between treatments. The light-saturated rate of photosynthesis (*A_sat_*) for the control was 27.6 μmol m^-2^ s^-1^, for PSL it was 27.74 μmol m^-2^ s^-1^ and for NSL it was slightly higher at 30.12 μmol m^-2^ s^-1^; however, these differences were not statistically significant (**Figure 2A**). The evening light response curves (after 12 hours, **Figure 2B**) revealed a decrease in the light-saturated rate of photosynthesis (*A_sat_*) for both the control and PSL-grown plants compared to morning measurements (Control: 20.66 μmol m^-2^ s^-1^; PSL: 20.74 μmol m^-2^ s^-1^; NSL: 31.37 μmol m^-2^ s^-1^), however, this drop was only statistically significant for the control. Notably, *A_sat_* was significantly higher (p< 0.05) in the NSL treatment at 31.37 μmol m^-2^ s^-1^ compared to the control and PSL (**Figure 2B**). The error bars indicate substantial variability among replicates compared to earlier measurements, suggesting potential differences in plant responses to light intensity during evening assessments.

**Figure 2C** illustrates the slope of the curve between data points and shows a linear decline in the rate of carbon assimilation up to ca. 475 μmol m^-2^ s^-1^. Beyond this threshold of 475 μmol m^-2^ s^-1^ increases in carbon assimilation greatly decrease, indicating diminishing returns with higher light intensities. These findings suggest that our target peak PPFD level for our initial sinusoidal experiments of 550 μmol m^-2^ s^-1^ exceeded the optimal requirement for photosynthesis.

**Figure 3:**
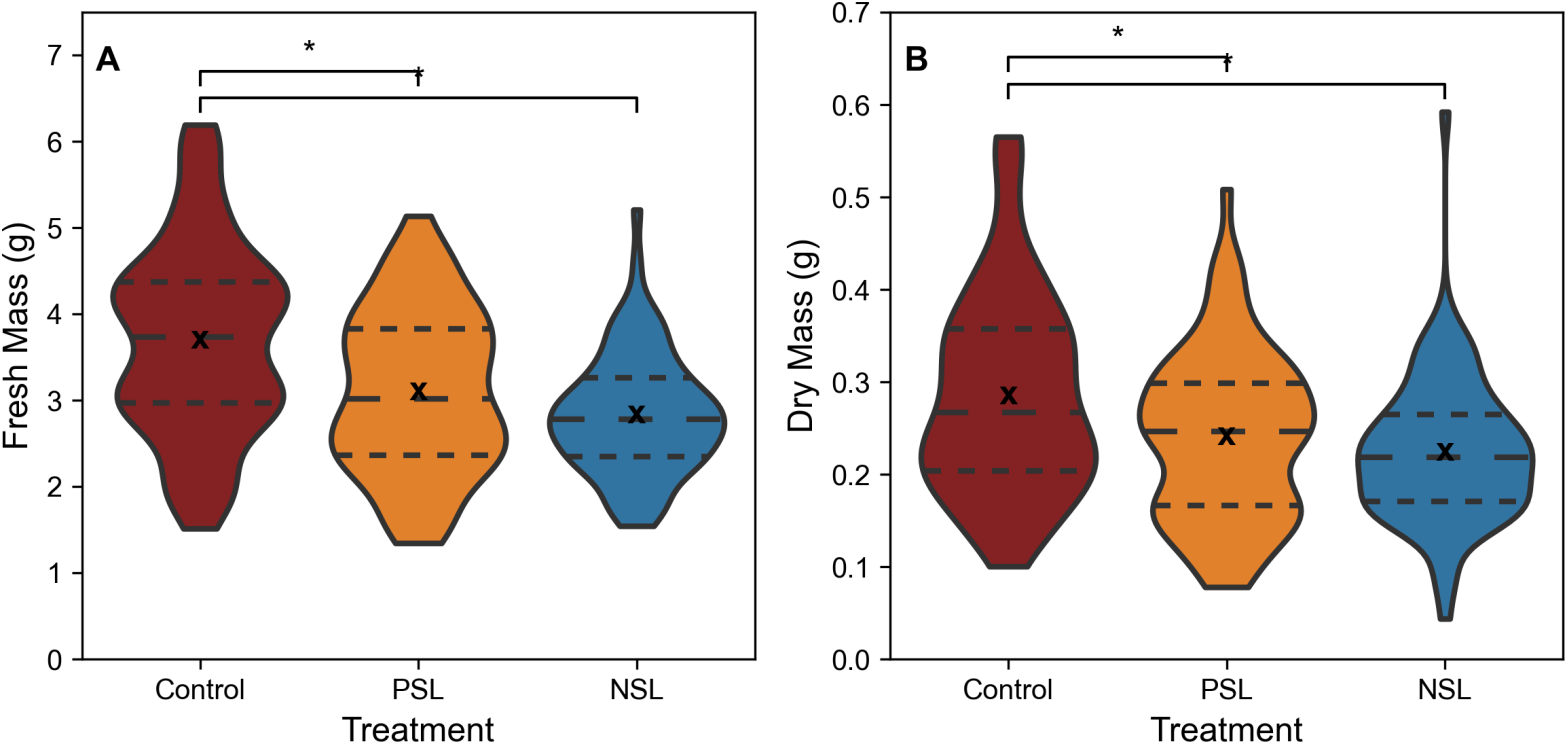
Fresh **(A)** and Dry mass **(B)** of rocket leaves, cut 1cm above the base of the plants from SL regimes. ‘*’ indicates non-parametric significance of p< 0.05. Long dashed line (— —) represents the median, small dashed line (– –) represents first and third quartile. ‘x’ indicates mean value. The width of the plot for each treatment represents the distribution of measured samples (n=76-84).

When comparing biomass, the control treatment exhibited significantly higher fresh (**Figure 3A**, median fresh mass 3.74 g, SD 1.11) and dry biomass (**Figure 3B**, median dry mass 0.27 g, SD 0.11) compared to the two other treatments (p<0.05, **Figures 3A, B**). The median fresh biomass for the NSL treatment was 2.78 g, (SD 0.70) and dry biomass 0.22 g (SD 0.08), while the PSL median fresh biomass was 3.02 g, (SD 0.94) and dry biomass 0.25 g, (SD 0.09). No significant differences in either fresh or dry biomass were apparent between the PSL and NSL treatments.

The electricity usage throughout the growing period was assessed for all treatments, revealing variations in consumption levels (Control: 0.926 kWh/day, PSL: 0.897 kWh/day, NSL: 0.885 kWh/day). Specifically, the PSL growing regime demonstrated a 3.1% reduction compared to the control, while the NSL treatment showed a 4.4% reduction in electricity usage (**Table 1**).

**Table 1:** Electricity use over 24h for each experimental light regime and percentage saving of each treatment relative to control.

Experiment	Light regimes	Electricity use (kWh)	Electricity saving (%)
Sinusoidal light regimes (SL)	Control (SW)	0.926	–
	PSL	0.897	3.1
	NSL	0.885	4.4
Capped sinusoidal regimes (CL)	Control (SW)	0.836	–
	CL250	0.831	0.6
	CL300	0.831	0.6
	CL400	0.854	-2.2

### Capped lighting regimes

**Figure 4:**
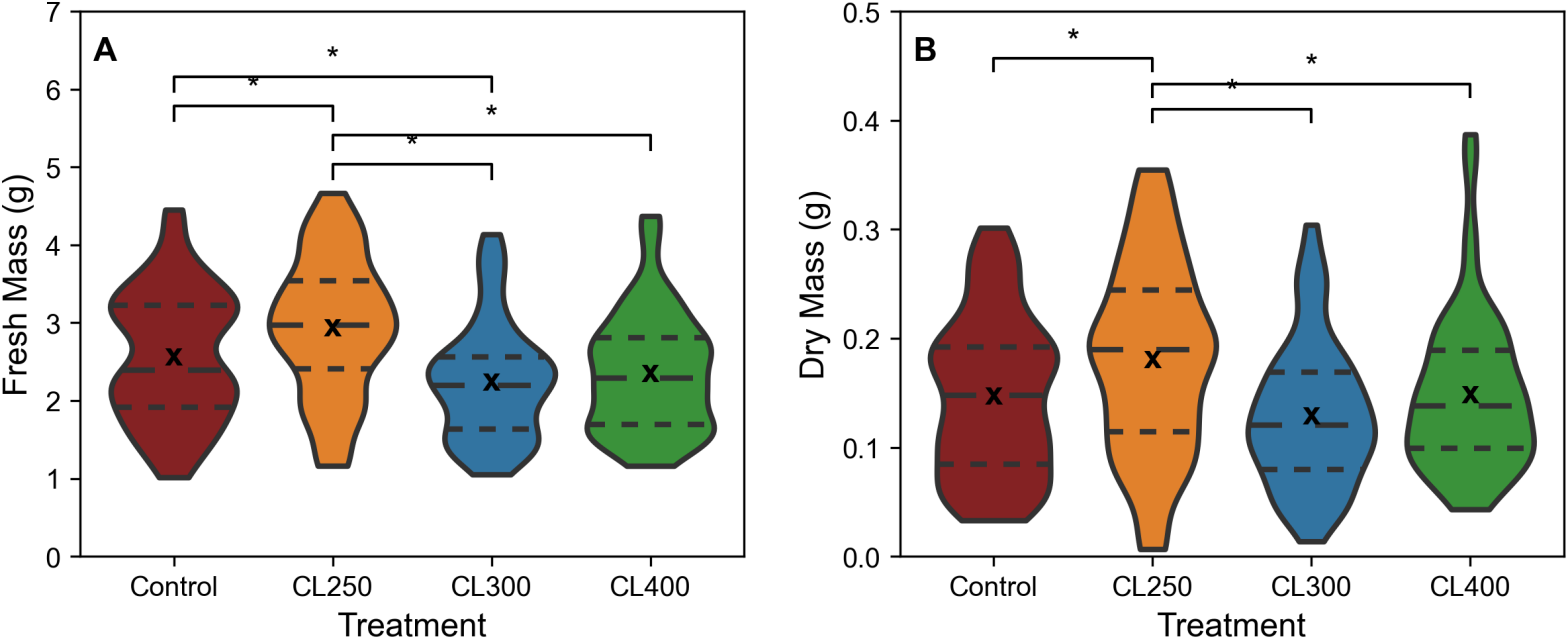
Fresh **(A)** and Dry mass **(B)** of harvested rocket leaves, cut 1cm above the base of the plant from CL regimes. ‘*’ indicates non-parametric significance of p< 0.05. Long dashed line (— —) represents the median, small dashed line (– –) represents first and third quartile. ‘x’ indicates mean value. The width of the plot for each treatment represents the distribution of measured samples (n=51-63).

Based on the data from **Figures 2** and **3**, suggesting PPFDs exceeding 475 μmol m^-2^ s^-1^ start to saturate photosynthesis, a new experiment was performed using sinusoidal light regimes that were capped at light intensities of either 250, 300 or 400 μmol m^-2^ s^-1^ (CL250, CL300 and CL400). Biomass comparisons indicated that the CL250 treatment exhibited a significantly higher fresh (median fresh mass 2.97 g) and dry biomass (median dry mass 0.19 g) compared to all other treatments (p<0.05; **Figures 4A, B**; control: median fresh mass 2.39 g, median dry mass 0.15 g, CL300: median fresh mass 2.20 g, median dry mass 0.12 g, CL400: median fresh mass 2.29 g, median dry mass: 0.14 g). The CL300 treatment displayed significantly lower fresh biomass than the control, but no other significant differences were apparent for either CL300 or CL400 compared to the control. In addition to producing a greater fresh and dry mass, the CL250 treatment *also* achieved this using less energy (ca. 0.6%) compared with the control (**Table 1**). Although the CL300 treatment also had an similar energy saving o, given the decrease in fresh biomass this would not be commercially relevant.

Photochemical quenching (*F_q_’*/*F_v_’*) and the maximum efficiency of PSII photochemistry (*F_v_’*/*F_m_’*) (with lower values indicative of higher NPQ) determined from chlorophyll fluorescence measurements for the first 5 h of the photoperiod for each treatment (**Supplementary Figure 1**) illustrated lower NPQ values for the CL250 treatment compared with others regimes, whilst *F_q_’*/*F_v_’* was maintained at a higher level indicating greater utilization of the end products (ATP and NADPH) of electron transport (ET) (**Supplementary Figure 1B**).

**Figure 5:**
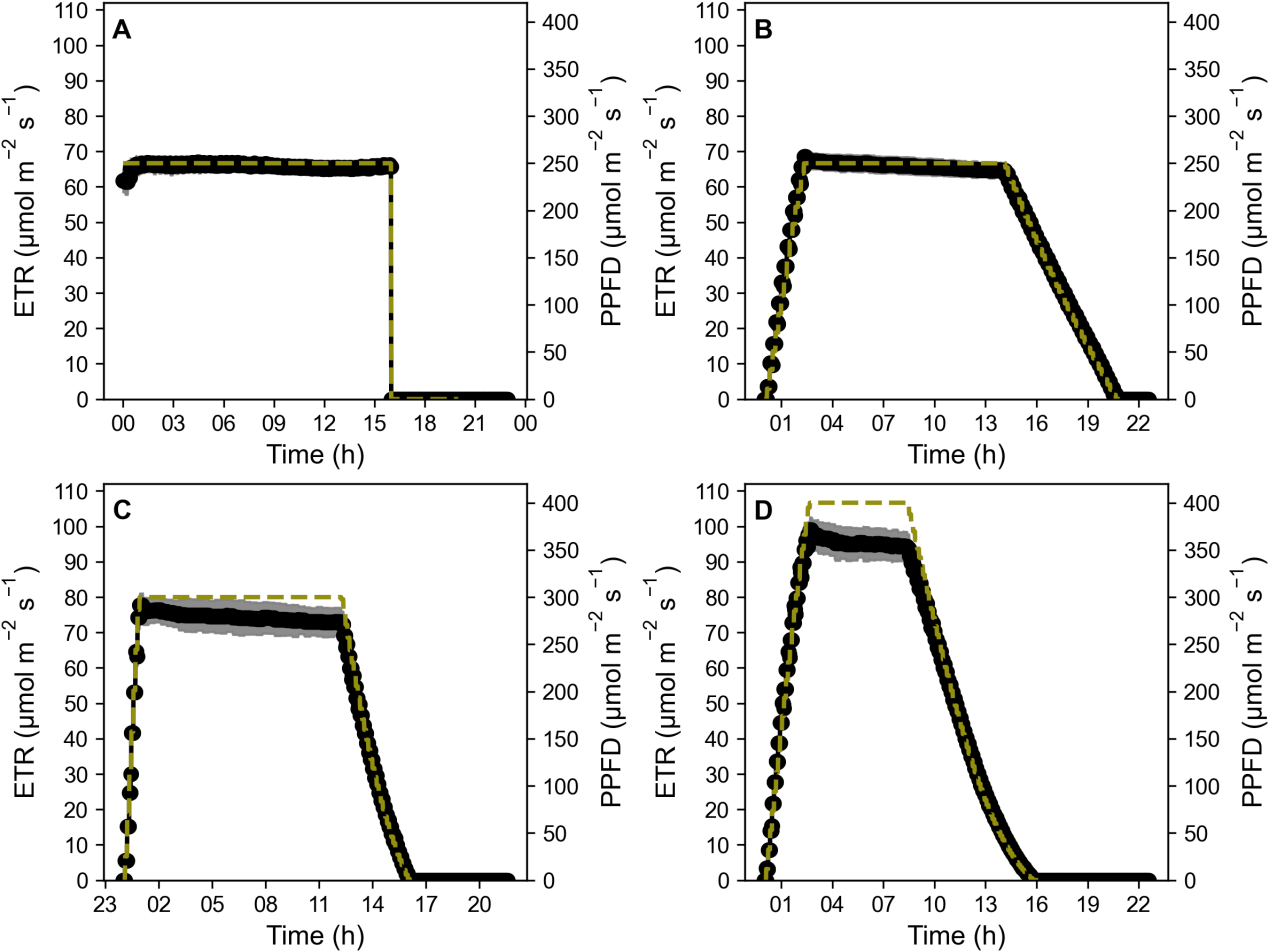
Diurnal electron transport rate (ETR) of Control treatment **(A)** CL250 **(B)**, CL300 **(C)** and CL400 **(D)** (black dots). Measurements were collected every 20 min. Yellow dashed lines (– –) represent PPFD levels. Error bars ± SD (n=5-8).

Diurnal Electron Transport Rate (ETR) (**Figure 5**) derived from measurements of photosynthetic efficiency showed a consistent trend of decreasing values over the period of constant light exposure. In the control treatment, this decrease only became apparent after 6-9 h into the lighting period. The drop equated to 2.3% from first stable measurement to end of photoperiod. However for the CL regimes, the decrease in ETR started almost immediately at the beginning of the constant light period. Moreover, the reduction in ETR was significantly higher in CL300 and CL400 regimes compared to the control and CL250 (Control: -2.3%; CL250: -3.6%; 300CL: -4.4%, p=0.04; CL400: -4.7%, p=0.03). No significant differences in percentage decrease of ETR were apparent between the control and CL250 treatment. This suggests that length of time spent at peak PPFD in higher capped treatments (CL300 and 400) led to a more rapid and substantial decline in PSII operating efficiency compared to the control treatment.

**Figure 6:**
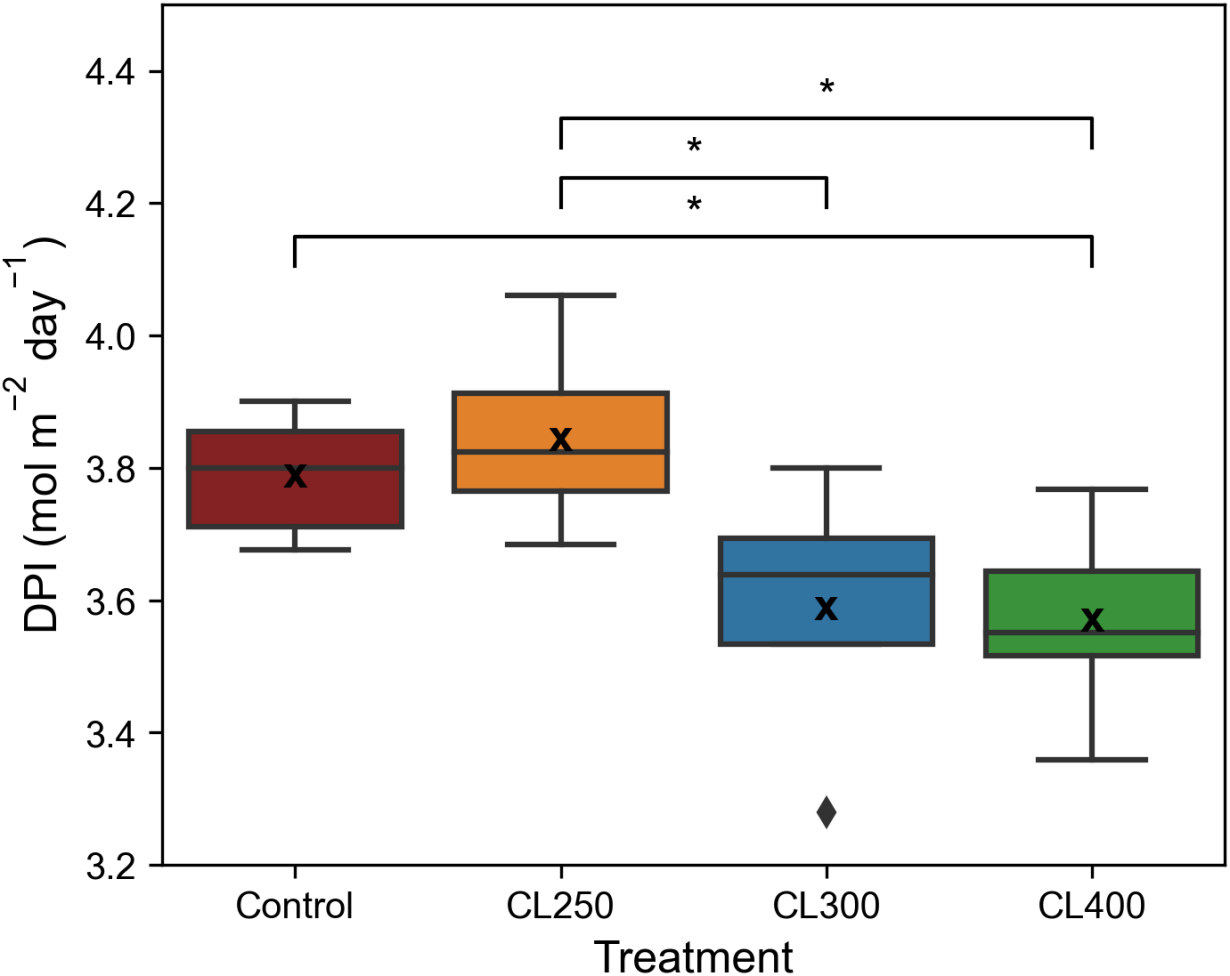
Daily photochemical index (DPI). ‘*’ indicates non-parametric significance of p< 0.05. Central line represents the median, outer lines of the box (– –) are the first and third quartile. ‘x’ indicates mean value (n=5-8).

The decrease in diurnal ETR was reflected in the median Daily Photochemical Integral (DPI), which showed that the median DPI for the control and CL250 treatment was significantly higher (p< 0.05, Control: 3.79 mol m^-2^ day^-1^, CL250: 3.84 mol m^-2^ day^-1^) than both the CL300 and CL400 treatments (CL300: 3.59 mol m^-2^ day^-1^, CL400 CL 3.57 mol m^-2^ day^-1^, **Figure 6**). These findings suggest that light levels below 300 μmol m^-2^ s^-1^ promoted higher diurnal electron transport rates compared to higher light intensities, highlighting the impact of light intensity on photosynthetic efficiency despite equal Daily Light Integral (DLI) values.

Electricity usage in the capped light treatments revealed a reduction in use in all CL regimes compared with the control (**Table 1**). Energy use for both CL250 and CL300 were 0.6% lower than the control, however CL400 caused a 2.2% increase in energy use, suggesting that the effectiveness of capped light regimes in improving energy efficiency is dependent on the light intensity, with a PPFD capped at 400 μmol m^-2^ s^-1^ (CL400) being less energy efficient.

## Discussion

In field conditions crops experience a range of different light intensities that change over the course of the day from dawn to sunset, which is modified by cloud cover, in addition to overlapping leaves from adjacent plants (Vialet-Chabrand et al., 2017). The spatial and temporal dynamic impact of these fluctuating conditions tends to lead to non-optimal heterogeneous photosynthetic and stomatal conductance (Pearcy, 1990; Lawson et al., 1998) with implications for photosynthetic induction and water use (Matthews et al., 2018; Lawson et al., 2012; Long et al., 2022). Therefore, in controlled cropping environments, growers often attempt to optimize conditions with the aim of keeping a constant light intensity throughout the photoperiod, using either supplementary lighting in systems that receive natural light, or using square wave light conditions where LEDs provide the only light for growth (Elkins and van Iersel, 2020a, 2020b). However, several studies have demonstrated that at the start of the light period photosynthetic induction can be a slow process, and depending on species, can take tens of minutes to become optimal (Kaiser et al., 2015; Lawson et al., 2012). The slow rate of photosynthetic induction can have significant impacts on carbon gain and plant biomass (Adachi et al., 2019; Kimura et al., 2020; Yamori et al., 2020; Deans et al., 2019; Salter et al., 2019) due to the slow activation of Calvin Benson cycle enzymes (Pearcy et al., 1996), including Rubisco (Carmo-Silva et al., 2015) and sluggish stomatal opening (Lawson et al., 2010; Deans et al., 2019). Furthermore, when plants experience a sudden and rapid change in light intensity this can lead to increased NPQ or photoinhibition (Sun et al., 2023). In addition, it is well established that photosynthetic carbon gain and photosynthetic efficiency decrease towards the end of the photoperiod (Pearcy, 1990; Vialet-Chabrand et al., 2017; Matthews et al., 2018) and NPQ varies over the diurnal period which also depends on lighting regimes (Lazzarin et al., 2024). These findings raise the possibility that optimizing lighting regimes to account for physiological changes could not only enhance plant growth in controlled environment agriculture (CEA) but also reduce energy inputs. Here, we employed a range of sinusoidal light regimes characterized by gradual increases and decreases in light intensity to identify optimal lighting conditions for indoor rocket cultivation. By carefully tailoring light exposure patterns to align with plant physiological responses, we aim to maximize growth efficiency and minimize energy consumption in indoor CEA settings. This approach highlights the importance of strategic light management in achieving sustainable and profitable crop production within controlled environments.

To assess the impact of light on physiology we measured photosynthesis as a function of light intensity. Whilst all light response curves followed the typical hyperbolic pattern in all treatments and at both time points within the photoperiod (i.e. after 4 hours and after 12 h) (**Figures 2A, B**), *A_sat_* was markedly reduced under square wave and PSL conditions after 12 h (**Figure 2B**), illustrating the previously reported drop in *A* over the photoperiod (Vialet-Chabrand et al., 2017; Matthews et al., 2018). Interestingly this reduction was not observed for the NSL regime, with a high *A_sat_* maintained at 12h, which may be due to a later start of the photoperiod in this treatment with maximum PPFD only reached after 12 h, compared to 4 h in the PSL regime and instantaneously in the control. It is likely that the later onset of the photoperiod in the NSL regime had not yet caused a decrease in sink capacity for the end products of electron transport (ATP and NADPH), as reflected in the observed decrease in *A* in the other treatments (Murchie and Lawson, 2013; Yamori, 2016). This reduction has previously been linked to feedback control on the Calvin Benson Cycle via sugar accumulation later in the diurnal period, possibly due to changes in gene expression of photosynthetic proteins (Paul and Foyer, 2001; Paul and Pellny, 2003). The resulting reduction in *A* reduces consumption of ATP and NADPH which would lead to an accumulation of ATP and NADPH and feedback to slow ETR.

Unexpectedly, both our sinusoidal growth regimes had negative impacts on accumulation of edible biomass compared to the control square-wave light regime (**Figure 3**). Although *A*_sat_ determined from the 12h light response curve in the NSL treatment was greater than the control, this not only represents a single time-point in the diurnal period, it also fails to take into account any feedback inhibition driven by the accumulation of photosynthate, as these plants had received a much lower accumulated light dose up to the point the measurements was taken. Quantum efficiency, as illustrated by the slope of the light response curves, dropped significantly after 475 μmol m^-2^ s^-1^ in all treatments (**Figure 2C**), a light intensity that was exceeded in both the PSL and NSL, which peaked at 550 μmol m^-2^ s^-1^ (**Figure 1**). This suggests that even though the DLI was identical in all treatments, plants grown in sinusoidal light regimes were unable to utilize light above the near saturated threshold of 475 μmol m^-2^ s^-1^ for carbon fixation. Therefore, the total amount of light that was actually used to drive photosynthesis was lower in these treatments compared to the control, reducing potential carbon gain and possibly causing the observed reduction in both fresh and dry mass.

Our capped light regimes were designed to address this by keeping the maximum PPFD below the 475 μmol m^-2^ s^-1^ saturation threshold for each treatment, whilst maintaining the initial ‘controlled’ induction of photosynthesis and decrease in intensity towards the end of the photoperiod. Results for both dry and fresh biomass in this experiment agree with findings from a number of similar studies that have shown a lower PPFD over a longer photoperiod to increase accumulation of edible biomass compared to higher growth light intensities (Elkins and van Iersel, 2020a; Palmer and van Iersel, 2020; Weaver and van Iersel, 2020). The CL250 regime resulted in the highest yield compared to the other capped treatments and, interestingly the control, in which plants were grown at the same PPFD but under a square wave regime (**Figure 4**). This indicates that further gains in biomass are possible by adjusting lighting regimes to gradually increase at the start of the photoperiod and gradually decrease towards the end, whilst also maintaining an appropriate maximum light intensity for photosynthesis. The gradual rise in intensity at the start takes into account the slow increase in stomatal conductance with increasing irradiance (Lawson et al., 2012; Lawson and Vialet-Chabrand, 2019), as well as the slow activation of photosynthetic enzymes, preventing possible photodamage (Sun et al., 2023) or significant dissipation of energy via non-photochemical quenching process (which is illustrated by the lower values of *Fv’*/*Fm’* **Supplementary Figure 1**). The higher values of *F_q_’*/*F_v_’* also suggest greater utilization of the end products of electron transport (ET), most likely as a result of greater carbon assimilation (**Supplementary Figure 1B**). The gradual decrease in light at the end matches the reduction in photosynthetic capacity towards the end of the photoperiod (**Figure 5**) (see Vialet-Chabrand et al., 2017; Matthews et al., 2018; Kaiser et al., 2015). Our results also illustrate the importance of considering the intensity of the “capped” or maximum light level, as although all caps were below the saturated value (of 475 μmol m^-2^ s^-1^) the reduced biomass in the CL300 and CL400 treatments is most likely the results of the significant drop in ETR over the constant proportion of the photoperiod compared to the control (**Figure 5**). The significantly higher median DPI of the control and CL250 treatment compared with the other two growth light regimes, confirm this by illustrating a greater overall ETR over the photoperiod at a lower light intensity (Elkins and van Iersel, 2020a).

Electricity costs associated additional lighting in horticulture and CEAs pose significant challenges for the industry (Elkins and van Iersel, 2020b). The sustainability of completely artificially illuminated growing environments is an additional concern (Bunge et al., 2022). Our analysis which integrates biomass production with energy usage, highlights the advantages of incorporating light regimes based on factors beyond just intensity and duration (i.e DLI). To date the majority of studies aiming to optimize lighting regimes for growth have overlooked dynamic lighting delivery strategies. Our findings presented here demonstrate that greater biomass (*ca.* 20%) with lower (0.6%) energy input is feasible through the optimization of photosynthetic efficiency across the *entire* photoperiod, by considering photosynthetic induction, maximum light intensity and associated decreased photosynthetic capacities to inform light recipes for CEAs. Future research should focus on refining lighting regimes using AI-generated recipes that incorporate physiological inputs and knowledge. Additionally, integrating sensor technology for real-time biofeedback based on plants’ needs is important for advancing this field (van Iersel et al., 2016). This approach holds promise for optimizing growth conditions in horticulture and CEA, leading to more efficient and sustainable practices, that will facilitate the industry in terms of profitability as well as carbon neutrality targets.

## Data Availability

The raw data supporting the conclusions of this article will be made available by the authors, without undue reservation.
