# Supplementary material for: Sinusoidal LED light recipes can improve rocket edible biomass and reduce electricity costs in indoor growth environments
Source: Front Plant Sci. 2024 Oct 3;15:1447368. doi: 10.3389/fpls.2024.1447368 (PMC11503027; doi:10.3389/fpls.2024.1447368)
Supplement: Supplementary file 1 [file Image1.pdf]

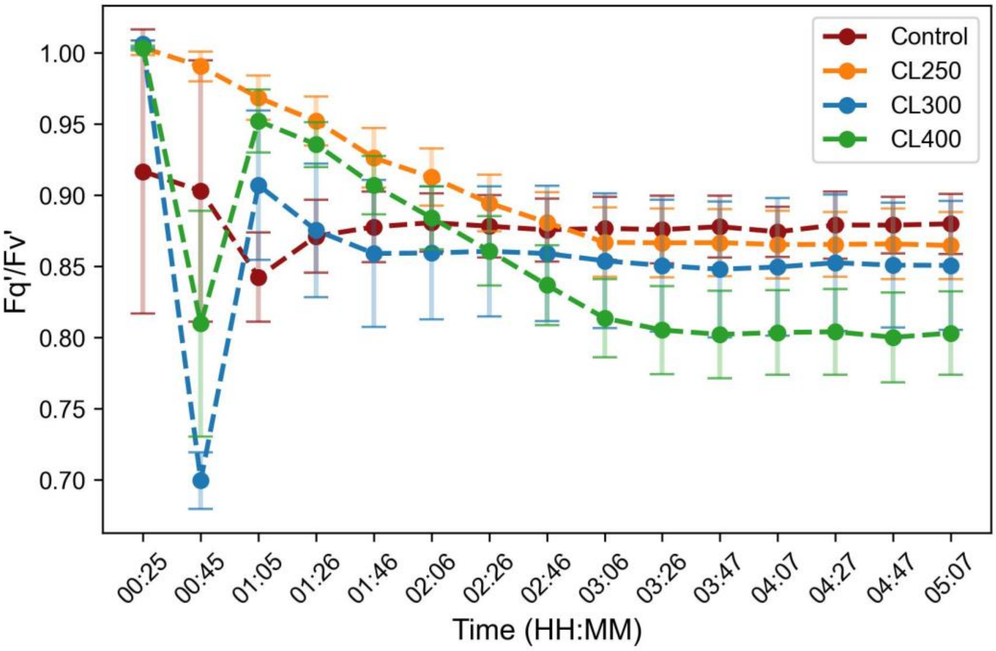

Supplementary Figure 1. Diurnal F_q'/F_v' of Control treatment (●) CL250 (●), CL300 (●) and CL400 (●). Measurements were collected every 20 min. Error bars ± SD (n=5-8).

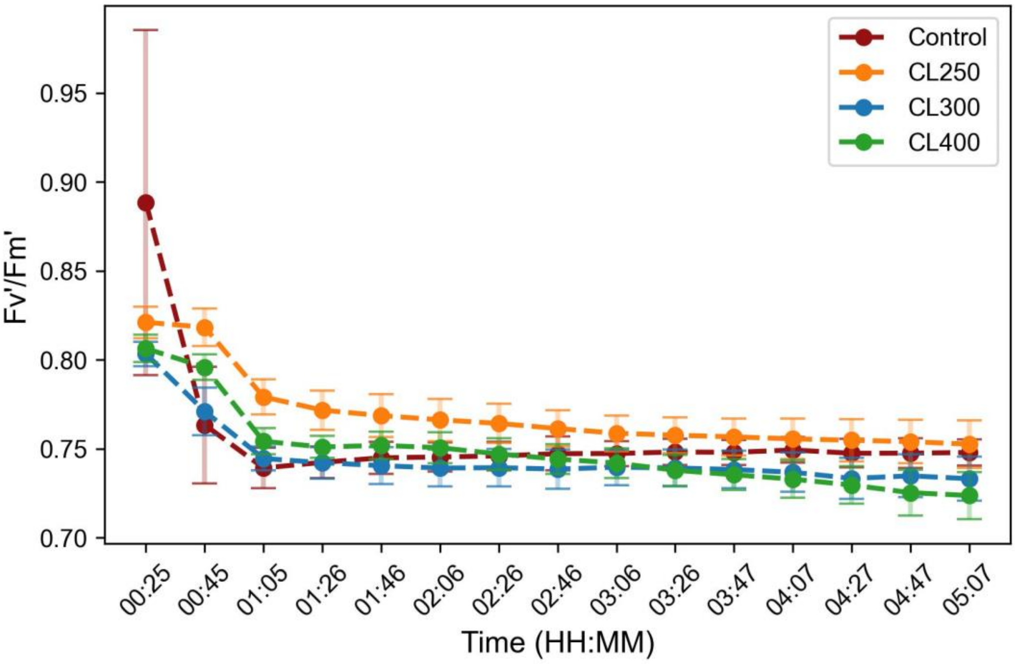

Supplementary Figure 2. Diurnal F_v'/F_m' of Control treatment (●) CL250 (●), CL300 (●) and CL400 (●). Measurements were collected every 20 min. Error bars ± SD (n=5-8).
